# Safety of the harmonic scalpel versus conventional “clamp-and-tie” approach for intraoperative bleeding control during thyroidectomy: a surgeon’s experience in 60 consecutive cases

**DOI:** 10.1186/s13037-026-00501-2

**Published:** 2026-07-11

**Authors:** Soniya Shankar Kalpana, G. Tilak Ramu, Balaji Durairaj

**Affiliations:** Department of General Surgery, SRM Medical College and Hospital & Research Centre, Faculty of Medicine and Health Sciences, SRM IST, Kattankulathur, Chengalpattu (Dt), Tamil Nadu India

**Keywords:** Thyroidectomy, Harmonic scalpel, Recurrent laryngeal nerve injury, Thyroid surgery, Surgical safety

## Abstract

**Background:**

Post-thyroidectomy complications including hypocalcemia, recurrent laryngeal nerve injury, and cervical hematoma remain important safety concerns. Although the harmonic scalpel improves operative efficiency, evidence regarding its impact on patient safety outcomes remains limited.

**Methods:**

In this prospective observational cohort study, 60 patients undergoing open thyroidectomy were allocated to harmonic scalpel (*n* = 30) or clamp and tie (*n* = 30) groups. The primary endpoints were safety outcomes, including hypocalcemia, recurrent laryngeal nerve injury, postoperative hematoma, surgical site infection, and composite complication rates. Secondary endpoints included operative time, intraoperative blood loss, drain output, postoperative pain, and length of hospital stay. Statistical significance was defined as *p* < 0.05.

**Results:**

Use of the harmonic scalpel was associated with a significant reduction in overall complications compared with the conventional technique (13.3% vs. 33.3%, *p* = 0.04). Transient hypocalcemia occurred less frequently in the harmonic scalpel group, accompanied by significantly higher postoperative calcium levels at 24 and 48 h. No recurrent laryngeal nerve injury or postoperative hematoma was observed in the harmonic scalpel group, although differences were not statistically significant. In addition to improved safety outcomes, harmonic scalpel use resulted in shorter operative duration, substantially reduced intraoperative blood loss, lower postoperative drain volumes, decreased pain scores, and reduced length of hospital stay.

**Conclusion:**

The harmonic scalpel was associated with improved operative efficiency and a lower overall complication burden without increasing major safety-related complications. Larger randomized multicenter studies are required to confirm these findings.

**Clinical trial number:**

Not applicable.

## Introduction

Thyroidectomy is one of the most commonly performed endocrine surgical procedures worldwide and remains the standard treatment for a wide range of benign and malignant thyroid disorders [1, 2]. While the procedure is generally safe in experienced hands, it is not without risk. Complications such as hypocalcemia, recurrent laryngeal nerve injury, and postoperative cervical hematoma continue to represent important patient safety concerns due to their potential impact on airway integrity, voice function, and overall recovery. Consequently, modern thyroid surgery has evolved with an increasing emphasis on minimizing preventable harm while maintaining oncological adequacy and functional preservation [1–3].

Achieving effective hemostasis is central to safe thyroid surgery. The conventional clamp-and-tie technique has long been regarded as the standard approach, relying on meticulous vessel identification, ligation, and dissection. Its reliability, accessibility, and well-established safety profile have made it a cornerstone of surgical training and practice [4]. However, this method is inherently labor-intensive, particularly in the highly vascular thyroid bed, and may require repeated tissue handling and instrument exchanges. These factors can contribute to increased intraoperative bleeding, prolonged operative duration, and reduced clarity of the surgical field, all of which may indirectly influence the risk of injury to critical structures [5].

In recent years, advances in surgical technology have introduced energy-based devices aimed at improving both efficiency and safety. Among these, the harmonic scalpel has gained considerable attention. This ultrasonic device enables simultaneous cutting and coagulation through high-frequency mechanical vibrations, resulting in protein denaturation and vessel sealing with relatively limited lateral thermal spread compared to conventional electrosurgical methods. By combining multiple steps into a single action, the harmonic scalpel has the potential to streamline dissection and reduce operative complexity [3, 6].

From a patient safety perspective, the theoretical advantages of the harmonic scalpel extend beyond efficiency. Improved hemostasis may enhance visualization of the operative field, facilitating more precise identification and preservation of vital structures such as the recurrent laryngeal nerve and parathyroid glands [7, 8]. In addition, reduced tissue trauma and fewer instrument exchanges may lower the likelihood of inadvertent injury to adjacent neurovascular and parathyroid structures [7, 9]. Several studies have reported shorter operative times, decreased blood loss, and reduced postoperative discomfort with the use of the harmonic scalpel; however, the extent to which these benefits translate into meaningful reductions in complication rates remains a subject of ongoing debate [3, 5, 6, 10, 11].

The use of harmonic scalpel technology in thyroid surgery is well established and has been reported for more than two decades, with early studies demonstrating its feasibility and safety in thyroidectomy procedures [4, 12]. More recent systematic reviews and meta-analyses have continued to support reductions in operative time, blood loss, and postoperative morbidity; however, evidence regarding its impact on recurrent laryngeal nerve injury and other major complications remains inconsistent across studies [3, 6, 11, 13, 14].

Importantly, the relationship between operative efficiency and surgical safety is complex. While shorter operative time is often considered desirable, it does not independently guarantee improved outcomes. Evidence suggests that operative duration alone may not be a definitive predictor of postoperative complications in thyroid surgery [15]. Rather, factors such as surgical precision, hemostatic control, and preservation of critical anatomical structures are likely to play a more direct role in determining patient safety outcomes [3].

Comparative studies and meta-analyses evaluating harmonic scalpel-assisted thyroidectomy versus conventional techniques have yielded mixed results. Although several studies have demonstrated reductions in operative time, blood loss, drain output, and postoperative pain [1, 10, 11, 16, 17], differences in major complications such as hypocalcemia, recurrent laryngeal nerve injury, and postoperative hematoma have not been consistently observed [3, 13, 14, 18].

Despite the growing body of literature supporting the use of energy-based devices in thyroid surgery, most studies have primarily focused on operative efficiency, whereas prospective real-world data specifically evaluating patient safety outcomes remain limited [3, 6, 11, 13, 14]. Therefore, the present study was undertaken to compare harmonic scalpel-assisted thyroidectomy with the conventional clamp-and-tie technique in open thyroidectomy, with particular emphasis on postoperative safety outcomes, including hypocalcemia, recurrent laryngeal nerve injury, postoperative hematoma, and overall complication rates. Secondary outcomes included operative efficiency and postoperative recovery parameters.

## Methods

This prospective observational cohort study was conducted in the Department of General Surgery at a tertiary care teaching institute to evaluate the impact of different hemostatic techniques on patient safety in open thyroidectomy. The study compared outcomes between patients undergoing thyroidectomy using the harmonic scalpel and those treated using the conventional clamp-and-tie technique.

Patients were enrolled consecutively, and all clinical, intraoperative, and postoperative data were collected prospectively to ensure accuracy and minimize recall bias. The study was specifically designed to assess complication rates and determine whether the choice of hemostatic technique influences surgical risk and patient safety outcomes. Ethical approval was obtained from the Institutional Ethics Sub-Committee (Human Studies). Written informed consent was obtained from all participants in their preferred language after a detailed explanation. Patient confidentiality was maintained through anonymization of data. The study was conducted and reported in accordance with the STROBE guidelines. Patient selection, eligibility assessment, allocation, follow-up, and final analysis are summarized in the study flow diagram (Fig. 2).

### Study setting and time-window

The study was conducted from August 2024 to November 2025 over a period of 16 months at a tertiary health care academic institution. All patients underwent standardized preoperative evaluation, operative management, and postoperative follow-up within the same center, ensuring continuity of care.

Standard perioperative protocols were followed for all patients, with particular emphasis on early detection and management of safety-related complications, including hypocalcemia, postoperative hematoma, and voice changes.

### Eligibility criteria

Patients aged 18 years or older who were scheduled to undergo open thyroidectomy, including hemithyroidectomy or total thyroidectomy, were eligible for inclusion in the study. Indications for surgery included benign thyroid nodules, multinodular goiter, and differentiated thyroid carcinoma. All participants provided informed consent before enrolment. Patients undergoing reoperative thyroid surgery, those with a history of previous neck irradiation, known bleeding or coagulation disorders, preoperative vocal cord dysfunction, pregnancy or lactation, or those deemed unfit for general anesthesia were excluded from the study. Patients with incomplete clinical data were also excluded from the final analysis.

### Sampling and sample size

A consecutive sampling strategy was adopted, whereby all patients meeting the eligibility criteria during the study period were included, ensuring a pragmatic approach while reducing the risk of selection bias. The required minimum sample size was estimated to be 56 based on an anticipated difference in postoperative complication rates between groups, assuming a significance level of 5% and a study power of 80%. To accommodate potential attrition, the final sample size was increased to 60 patients, with 30 patients included in each study group.

### Study groups and allocation

Participants were divided into two groups based on the hemostatic technique used:

- Harmonic Scalpel Group (*n* = 30): Surgery performed using the harmonic scalpel for vessel sealing and dissection.
- Conventional Group (*n* = 30): Surgery performed using the clamp-and-tie technique.

All thyroidectomies included in the study were performed by a single consultant surgeon experienced in thyroid surgery. The choice of hemostatic technique was determined by the availability of the harmonic scalpel on the day of surgery and institutional resource considerations. Patients were enrolled consecutively, and no randomization was performed. Cases in which the harmonic scalpel was available were included in the harmonic scalpel group, whereas cases performed using conventional vessel ligation techniques were included in the clamp-and-tie group. No crossover between groups occurred. Baseline demographic and clinical characteristics were compared to assess group comparability. At the end of the study period, 30 eligible patients had undergone thyroidectomy using the harmonic scalpel and 30 using the conventional clamp-and-tie technique, constituting the final study groups. The detailed patient selection and allocation process is illustrated in Fig. 2.

### Outcome measures

The primary outcome measures focused on complications directly related to patient safety. These included postoperative hypocalcemia, assessed using serum calcium levels measured at 24 and 48 h postoperatively; recurrent laryngeal nerve injury, assessed clinically during the postoperative period and confirmed by video laryngoscopy on postoperative day 7; postoperative cervical hematoma; surgical site infection; and overall postoperative complication rate. Secondary outcome measures included operative time recorded in minutes, estimated intraoperative blood loss, postoperative drain output measured daily with drain removal performed once the output was less than 10 mL, postoperative pain assessed using the Visual Analog Scale on postoperative days 1, 3, and 7, and duration of hospital stay measured in days. Potential confounders, including age, sex, indication for surgery, and extent of thyroidectomy, were recorded. A composite safety score was calculated as the sum of these predefined postoperative complications, with each complication assigned one point, resulting in a total score ranging from 0 to 4 for each patient. This composite measure was used to provide a cumulative assessment of postoperative risk, as individual complications occur relatively infrequently and may not independently demonstrate statistically significant differences. By combining clinically relevant complications, this approach allows for a more sensitive evaluation of overall patient safety. All individual components of the composite outcome were also analyzed and reported separately.

All patients were followed up during the postoperative hospital stay and reassessed on postoperative day 7 for evaluation of complications, including hypocalcemia, recurrent laryngeal nerve injury, hematoma, and surgical site infection. Vocal cord function was assessed clinically and confirmed using laryngoscopic evaluation where indicated.

### Surgical procedure and postoperative monitoring

**Fig. 1:**
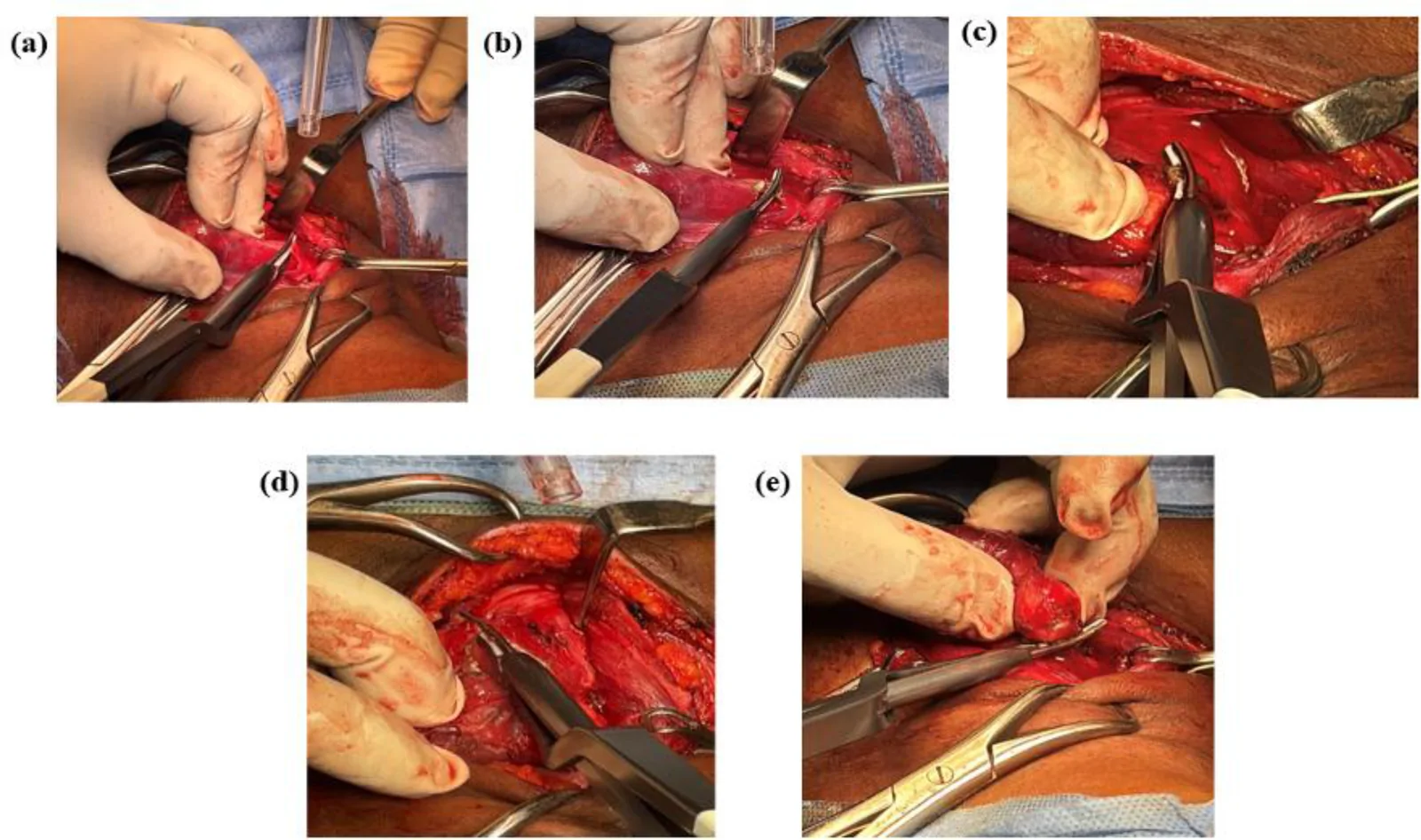
Intraoperative thyroidectomy images showing Harmonic scalpel-assisted dissection: (**a**) thyroid exposure and dissection, (**b**) thyroid lobe dissection, (**c**) inferior pole dissection, (**d**) capsular dissection with thyroid elevation, and (**e**) thyroid lobe mobilization and delivery

All procedures were performed by the same consultant surgeon under general anesthesia using a standardized open thyroidectomy technique. Figure 1 illustrates the surgical procedure; a standard transverse cervical incision (4–6 cm) was made. After elevating subplatysmal flaps, the strap muscles were separated and retracted to expose the thyroid gland. The key steps of vessel identification (superior, middle, and inferior thyroid vessels) and division were performed using the designated technique for each group. The recurrent laryngeal nerve was identified and meticulously preserved under direct vision throughout the procedure. Upon completion of the hemithyroidectomy or total thyroidectomy, a Romovac suction drain was placed in the thyroid bed, and the wound was closed in layers. Postoperatively, patients were closely monitored for early detection of complications. Serum calcium levels were measured at 24 and 48 h. Voice assessment was performed clinically, and video-laryngoscopy was conducted on postoperative day 7. Drain output, pain scores, and duration of hospital stay were recorded systematically.

### Bias and validity considerations

Consecutive sampling and prospective data collection were used to minimize selection and recall bias. Standardized surgical and postoperative protocols ensured uniform assessment across both groups. Although allocation was not randomized, this pragmatic design reflects routine clinical practice and enhances the external validity of the findings. Potential allocation bias arising from the non-randomized design and device availability was acknowledged; however, baseline comparability between groups reduced its potential impact. The use of a single-surgeon cohort minimized variability in surgical technique and operative decision-making, thereby improving procedural consistency across study groups.

### Data collection and statistical analysis

Data collection was carried out prospectively using a standardized case record form, capturing demographic information, intraoperative details, and postoperative outcomes. Recurrent laryngeal nerve function was specifically evaluated using video laryngoscopy on postoperative day 7.

Statistical analysis was conducted using SPSS software (version 26.0; IBM Corporation, Armonk, NY, USA). Continuous variables were summarized as mean ± standard deviation, while categorical variables were presented as frequencies and percentages. Group comparisons were performed using the independent samples t-test for continuous data and the Chi-square test or Fisher’s exact test for categorical variables, depending on suitability. A p-value of less than 0.05 was considered statistically significant. No missing data were observed for the primary outcomes, and complete follow-up was achieved for all participants. Where relevant, differences between groups were reported along with 95% confidence intervals. All measurements were obtained using consistent methods across both groups to maintain comparability.

## Results

**Fig. 2:**
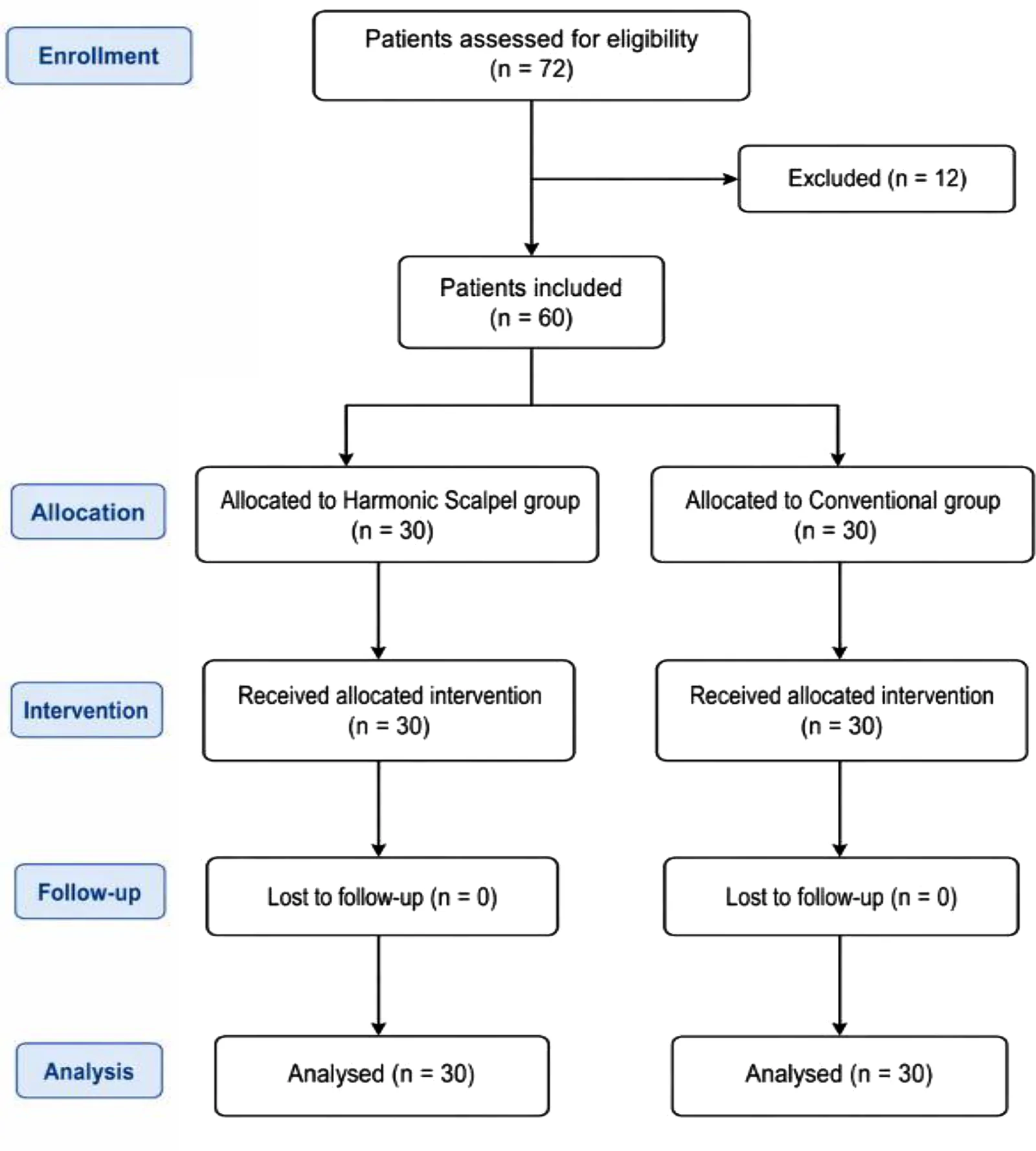
Flow diagram showing patient assessment, allocation, follow-up, and analysis

A total of 72 patients were assessed for eligibility during the study period, of whom 12 were excluded due to not meeting the inclusion criteria or declining participation. The remaining 60 eligible patients were included, comprising 30 patients who underwent thyroidectomy using the harmonic scalpel and 30 who underwent the conventional clamp-and-tie technique. The composite safety score was compared between groups using the independent samples t-test or Mann–Whitney U test, depending on the normality of distribution. All participants completed follow-up, and no data were missing for any outcome variables. The patient flow through the study is illustrated in Fig. 2.

Baseline demographic and clinical characteristics were comparable between the two groups, with no significant differences in age or sex distribution, and all patients were followed for a minimum of 7 postoperative days for assessment of complications. Use of the harmonic scalpel was associated with significantly improved operative efficiency, with a shorter mean operative duration compared to the conventional technique (68.2 ± 12.1 vs. 88.5 ± 14.3 min; *p* < 0.001).

Intraoperative blood loss was also significantly reduced in the harmonic scalpel group (72.5 ± 18.4 mL vs. 128.3 ± 24.6 mL; *p* < 0.001), along with consistently lower postoperative drain output across the first two postoperative days (*p* < 0.001). Patients in the harmonic scalpel group experienced a shorter hospital stay (3.1 ± 0.8 vs. 4.2 ± 1.1 days; *p* = 0.002), reflecting faster recovery. With respect to patient safety outcomes, the incidence of individual complications such as transient hypocalcemia, recurrent laryngeal nerve injury, postoperative hematoma, and surgical site infection did not differ significantly between the two groups. However, the overall complication rate was lower in the HS group (13.3%) compared to the clamp and tie group (33.3%), suggesting a potential improvement in the overall safety profile. Postoperative serum calcium levels were consistently higher in the HS group at both 24 and 48 h, with a corresponding reduction in clinically evident hypocalcemia. Mean postoperative drain output on postoperative day 1 was 32.4 ± 10.5 mL in the harmonic scalpel group compared with 56.7 ± 15.2 mL in the conventional group (*p* < 0.001). Corresponding values on postoperative day 2 were 18.6 ± 7.8 mL and 36.5 ± 12.1 mL, respectively (*p* < 0.001). No cases of postoperative hematoma were observed in the harmonic scalpel group, whereas isolated cases occurred in the conventional group. Recurrent laryngeal nerve injury rates were low in both groups, with no permanent nerve injury reported in the harmonic scalpel group. Postoperative recovery parameters also favored the harmonic scalpel group, with lower pain scores recorded on postoperative days 1 and 3, as well as reduced drain output, indicating less tissue trauma and improved hemostasis. These factors likely contributed to earlier mobilization and shorter hospitalization. The incidence of postoperative complications, including hypocalcemia, recurrent laryngeal nerve injury, hematoma, and surgical site infection, was comparable between the two groups. Follow-up was complete for all patients up to postoperative day 7, and outcome data were available for the entire study population.

Given the low frequency of individual complications, statistically significant differences were not observed for isolated outcomes. However, when assessed using a composite safety score, a reduction in cumulative complication burden was noted in the harmonic scalpel group.

A detailed comparison of baseline characteristics, operative parameters, and postoperative outcomes is presented in Table 1.

**Table 1:** Comparison of baseline characteristics, operative parameters, and patient safety outcomes between harmonic scalpel and conventional clamp-and-tie thyroidectomy

Parameter	Harmonic Scalpel (*n* = 30)	Clamp & Tie (*n* = 30)	Mean Difference	95% Confidence Interval	*p*-value
Age (Years)	42.6 ± 11.3	44.1 ± 10.8	− 1.5	−7.2 to 4.2	0.59
Female (%)	22 (73.3%)	21 (70.0%)	3.3%	−19% to 25%	0.59
Operative time (min)	68.2 ± 12.1	88.5 ± 14.3	-20.3	-27.1 to -13.5	< 0.001
Intraoperative blood loss (mL)	72.5 ± 18.4	128.3 ± 24.6	-55.8	-66.7 to-44.9	< 0.001
Postoperative drain output Day 1 (mL)	32.4 ± 10.5	56.7 ± 15.2	-24.3	-30.8 to -17.8	< 0.001
Postoperative drain output Day 2 (mL)	18.6 ± 7.8	36.5 ± 12.1	−17.9	−23.2 to − 12.6	< 0.001
Hospital stay (days)	3.1 ± 0.8	4.2 ± 1.1	−1.1	−1.6 to − 0.6	0.002
Transient hypocalcemia	2 (6.7%)	5 (16.7%)	-10%	-27% to 7%	0.23
Recurrent laryngeal nerve injury	0	1 (3.3%)	-3.3%	-10% to 3%	0.31
Postoperative hematoma	0	1 (3.3%)	-3.3%	-10% to 3%	0.31
Surgical site infection	1 (3.3%)	2 (6.7%)	-3.4%	-14% to 7%	0.55
Overall complication rate	4 (13.3%)	10 (33.3%)	-20%	-40% to -1%	0.04

Note: Mean difference calculated as Harmonic Scalpel minus Clamp-and-Tie. Continuous variables are presented as mean ± standard deviation; categorical variables as number (%)

Analysis of composite outcomes demonstrated that a higher proportion of patients in the harmonic scalpel group experienced no postoperative complications. The mean composite safety score was also significantly lower in this group, indicating a reduced cumulative complication burden (Table 2).

**Table 2:** Composite patient safety outcomes

Parameter	Harmonic Scalpel (*n* = 30)	Clamp & Tie (*n* = 30)	*p*-value
Patients with no complications	26 (86.7%)	20 (66.7%)	0.04
Patients with ≥ 1 complication	4 (13.3%)	10 (33.3%)	0.04
Mean safety score (0–4) *	0.20 ± 0.48	0.50 ± 0.73	0.03

*Safety score calculated as the sum of: hypocalcemia, recurrent laryngeal nerve injury, hematoma, Surgical site infection (1 point each)

## Discussion

Despite significant advances in thyroid surgery, postoperative complications continue to pose important challenges to patient safety. Several of these complications are influenced by potentially modifiable intraoperative factors, including inadequate hemostasis, reduced operative field clarity, repeated tissue manipulation, and difficult dissection around vital structures. In view of this, the present study aimed to examine whether use of the harmonic scalpel could help minimize overall surgical risk by providing better intraoperative control and lowering the incidence of postoperative complications. Identifying modifiable intraoperative factors that can reduce these risks remains a key priority in modern surgical practice [9, 19].

In this study, baseline demographic and clinical characteristics were well balanced between the two groups, minimizing potential confounding and strengthening the internal validity of the findings. Comparable distributions of age, sex, and surgical indications suggest that the observed differences are likely attributable to the technique itself rather than patient-related variables. Similar methodological rigor has been reported in prior randomized and comparative studies evaluating energy-based devices in thyroid surgery, where improvements in operative efficiency and perioperative outcomes were demonstrated independent of baseline characteristics [2, 20–23]. Meta-analyses have further supported these findings, showing consistent reductions in operative time and intraoperative blood loss with the harmonic scalpel without compromising safety outcomes [7, 17].

A key observation in this study was the significant reduction in operative duration associated with the harmonic scalpel. This finding aligns with existing literature demonstrating that ultrasonic devices streamline dissection by combining vessel sealing and division, thereby reducing the need for repeated instrument exchanges and improving surgical flow [7, 11, 17, 24, 25]. However, it is important to interpret reduced operative time within a broader patient safety framework. Evidence from the prolonged duration of surgery is not a risk factor for postoperative complications in patients undergoing total thyroidectomy, indicating that operative duration alone is not an independent predictor of postoperative complications in thyroid surgery [8]. Rather, outcomes are more closely linked to the quality of dissection, adequacy of hemostasis, and preservation of critical structures. In this context, the shorter operative times observed with the harmonic scalpel likely reflect improved efficiency without compromising safety.

From a patient safety standpoint, the observed reduction in intraoperative blood loss and postoperative drain output may offer benefits that extend beyond shorter operative time. Improved hemostasis helps maintain a clearer surgical field, which can facilitate safer identification and preservation of critical structures such as the recurrent laryngeal nerve and parathyroid glands. In addition, reduced need for repeated instrument handling and tissue manipulation may help limit overall operative trauma. Taken together, these factors indicate that the harmonic scalpel may contribute to safer surgical dissection and lower intraoperative risk, rather than serving only as a tool for procedural efficiency. The harmonic scalpel also demonstrated superior hemostatic performance, as evidenced by significantly lower intraoperative blood loss and reduced postoperative drain output. Effective hemostasis is particularly important in thyroid surgery, where even minor bleeding can obscure critical anatomical landmarks and increase the risk of inadvertent injury. Improved visualization of the surgical field facilitates safer identification and preservation of the recurrent laryngeal nerve and parathyroid glands, which are essential for maintaining functional outcomes [7, 26]. These findings are consistent with previous studies demonstrating that ultrasonic energy devices provide reliable vessel sealing with minimal thermal spread, thereby enhancing surgical precision [11, 17, 24, 26, 27].

An important observation in this study is that given the low incidence of individual complications, the study may be underpowered to detect statistically significant differences in isolated outcomes. Individual complications did not differ significantly between groups, the overall complication rate was reduced in the harmonic scalpel cohort. This finding underscores the concept that improvements in surgical safety may occur through incremental reductions across multiple domains, rather than dramatic changes in a single complication. In this context, composite outcomes may better reflect the real-world impact of intraoperative techniques on patient safety. While individual complications may be infrequent and difficult to distinguish statistically in smaller cohorts, the combined reduction in adverse events highlights the importance of incremental improvements across multiple domains of surgical care [7, 11, 17, 28].

The findings related to hypocalcemia are particularly relevant, as postoperative calcium imbalance remains one of the most common complications following thyroidectomy. Although differences in clinically apparent hypocalcemia did not reach statistical significance, patients in the harmonic scalpel group demonstrated consistently higher postoperative calcium levels. This may reflect better preservation of parathyroid vascularity and reduced mechanical or thermal injury during dissection. Previous studies have similarly reported improved postoperative parathyroid hormone levels with the use of ultrasonic devices, supporting the hypothesis that precise energy delivery may contribute to improved biochemical outcomes [9, 23].

Recurrent laryngeal nerve injury remains a critical concern due to its impact on voice and quality of life and the potential need for complex reconstructive procedures in selected cases [8, 29]. In this study, recurrent laryngeal nerve injury rates were low in both groups, with no permanent nerve injury observed in the harmonic scalpel cohort. Although the incidence of recurrent laryngeal nerve injury was numerically lower in the harmonic scalpel group, the study was not powered to detect significant differences in this relatively infrequent complication. A clearer operative field, reduced bleeding, and fewer instrument exchanges may contribute to safer dissection around the nerve. These findings are consistent with prior analyses suggesting that energy-based devices, when used appropriately, do not increase the risk of recurrent laryngeal nerve injury and may offer protective advantages in certain settings [13, 14, 17].

Postoperative hematoma represents a potentially life-threatening complication due to the risk of airway compromise. The absence of hematoma in the harmonic scalpel group, compared with isolated cases in the conventional group, underscores the importance of meticulous hemostasis in ensuring patient safety. Even small reductions in bleeding risk can translate into meaningful improvements in postoperative outcomes, particularly in high-risk anatomical regions such as the neck [7, 26, 27].

Beyond complication rates, the harmonic scalpel was associated with improved postoperative recovery, including lower pain scores, reduced drain output, and shorter hospital stay. These findings suggest that reduced tissue trauma and improved intraoperative control may facilitate faster recovery and earlier mobilization. From a systems perspective, shorter hospital stays and improved efficiency may contribute to better resource utilization, particularly in high-volume centers and resource-constrained settings [7, 11, 17, 24, 25]. In addition, reduced operative duration may improve operating room utilization, decrease anesthesia exposure, and potentially reduce healthcare costs, which may be particularly relevant in high-volume endocrine surgery units.

An important aspect of the present study is the use of a composite safety score to evaluate cumulative complication burden. While individual complications such as hypocalcemia, recurrent laryngeal nerve injury, and postoperative hematoma were infrequent and did not differ significantly between groups, the composite outcome provided a more sensitive measure for detecting differences in overall patient safety. Although all components were equally weighted, which may not fully reflect differences in clinical severity, this simplified approach enhances interpretability and allows for a pragmatic assessment of overall risk in routine clinical practice.

Despite these strengths, certain limitations must be acknowledged. The relatively small sample size also limits statistical power for detecting differences in uncommon complications such as recurrent laryngeal nerve injury and postoperative hematoma. The non-randomized allocation of patients based on harmonic scalpel availability introduces the possibility of selection bias, although baseline demographic and clinical characteristics were comparable between groups. While this pragmatic approach reflects real-world practice, residual confounding cannot be completely excluded. In addition, the single-center design may affect generalizability. Therefore, the findings should be interpreted as hypothesis-generating and require confirmation in larger, multicenter randomized trials. Objective evaluation of voice outcomes using standardized tools was also not performed, which could provide additional insights into functional recovery [9, 19].

Overall, the findings of this study suggest that the harmonic scalpel offers meaningful advantages in operative efficiency and may contribute to an improved safety profile in open thyroidectomy. By enhancing hemostasis, reducing tissue trauma, and facilitating precise dissection, it supports a safer surgical environment without increasing complication risk. These benefits, combined with improved recovery parameters, support its integration into contemporary thyroid surgery practice, particularly in settings where both patient safety and resource optimization are critical considerations [7, 8, 11, 17, 25, 30].

## Conclusion

The findings of this prospective study suggest that the use of the harmonic scalpel in open thyroidectomy is associated with improved operative efficiency and a reduction in overall complication burden. Although individual complication rates were comparable, the observed decrease in composite adverse outcomes indicates a potential cumulative benefit in surgical safety. These findings support the concept that hemostatic technique selection may function as a modifiable intraoperative risk-reduction strategy within broader patient safety frameworks in thyroid surgery. Further large-scale randomized studies are required to validate these findings and define their role in routine clinical practice.

## Data Availability

No datasets were generated or analysed during the current study.
